# Supplementary material for: “Behavioural and metabolic risk factors of cardiovascular diseases among post-menopausal women: A cross-sectional study in Itahari sub-metropolitan city of Nepal”
Source: PLoS One. 2024 Nov 14;19(11):e0310725. doi: 10.1371/journal.pone.0310725 (PMC11563358; doi:10.1371/journal.pone.0310725)
Supplement: Attachment — Submitted filename: Responses to reviewer.docx [file pone.0310725.s001.docx]

Date: 11/13/2023

To Plos One,

**Subject:- Responses to reviewer**

Dear Sir/ Maam,

At first we would like to thank for your review and feedback to our work. As per the suggestions and feedbacks changes have been made to article. Questions raised during review along with answers are given below.

Thank you for stating the following financial disclosure: "Unfunded"

At this time, please address the following queries:

a) Please clarify the sources of funding (financial or material support) for your study. List the grants or organizations that supported your study, including funding received from your institution.

- None. Self management done by authors.

b) State what role the funders took in the study. If the funders had no role in your study, please state: “The funders had no role in study design, data collection and analysis, decision to publish, or preparation of the manuscript.”

- “The funders had no role in study design, data collection and analysis, decision to publish, or preparation of the manuscript.” As there was no funder for this study.

  c) If any authors received a salary from any of your funders, please state which authors and which funders.
- None of authors received salary from funders.
  d) If you did not receive any funding for this study, please state: “The authors received no specific funding for this work.”
- “The authors received no specific funding for this work.”

3. Thank you for stating the following in your Competing Interests section:  "No"

"The authors have declared that no competing interests exist.",

5. Review Comments to the Author

Reviewer #1: GENERAL:
1. Please write the full form of abbreviation for the first time in the text: **Done**
2. Please spell the single digit in the text (eg. Three for 3): **Done**

INTRODUCTION:
1. The fourth paragraph looks too lengthy and redundant. Please make it shorter and concise by including the main findings of different studies; rather than describing individual studies separately

**Changes as per feedback has been made.**

METHODS:
1. The formula for sample size calculation is n = Z2pq/e2; not I2 (where e2 is the margin of error) **: Done**
2. The sampling method that you have described does not look like simple random sampling **: Done**

4. You have mentioned that “Explanatory variables showing p value less than 0.2 in bivariate analysis were entered into logistic model for multivariate analysis.” Could you please explain why this was done? Why was 0.2 taken as a level of significance at this point, and 0.05 while doing logistic regression later??

-At p-value 0.05 very few factors were significant on bivariate analysis. By taking variables with p – value 0.02 into multivariate analysis factors which are actually significant can be seen in logistic analysis after adjusting confounding effects of other variabls. P value 0.05 is standard value to consider significant so on multivariate analysis 0.05 p value was considered.

RESULTS:
1. Please mention below each table how p-value was obtained and its level of significance

- Bivariate analysis through chi-square test and level of significance is 0.05 for both bivariate and logistic analysis.

DISCUSSION:
1. Please mention the possible limitations of your study, and measures taken to overcome those limitations

-Mentioned

Reviewer #2: Dear authors,
• Authors have used STEP III as well (e.g lipid profile).
• Sampling technique is wrong. Authors have used multistage cluster random sampling, not simple random sampling.
Introduction:
• In fourth paragraph, study from Bangladesh came suddenly. Please either use general findings or describe studies from Nepal.
Method:
• Please change the name of methodology to method.
• This was not simple random sampling technique
• Please change inadequate intake of fruits and vegetable intake instead of “Low fruits and vegetable intake”.
• One entire section is missing. How the outcomes were measured (e.g BP, salt intake, BMI etc.). Data is missing on how many times the BP was measured.
• Method does not describe about how data was collected for lipid profile. There is no information about blood sample intake and biochemical measurement. I would recommend to describe the data collection techniques properly, to be able to replicate the findings.
• I do not understand why authors have missed data of blood sugar, diabetes mellitus.
Result:
• What is the rationale for determining the determinants of obesity/overweight and raised blood pressure only?
• Could you provide the data on age of menopause. Early menopause is one of the risk factor of CVD
Discussion:
• Please write a brief summary of the key findings of the study at first paragraph.
• In some of the paragraphs, authors have missed the citation (e.g This finding highlights the need to regulate price of fruits and vegetables by government sectors and also to develop job opportunity to rise financial status of people (?), low consumption of fruits and vegetables among the respondents and also changing lifestyle because of urbanization and lack of availability of organic fruits and vegetables in the market (?).
• Discussion section is too long.
• Please add a paragraph on the implications for policy and future research recommendation.
• What are the limitations of the study?

Language edition
• Language needs to be corrected (e.g community-based CVDs risk factors prevention and management among postmenopausal women is the need of the day (?)

- **Correction as per feedback done.**

Reviewer #3: Throughout the manuscript, there are missing punctuations, make fonts and sizes uniform, and needs grammatical refinement.

Title: "risk" must start with capitalize letter.

Introduction: In any articles, fullform of words need to be mentioned prior using the abbreviations, need references for first and second lines in 5th paragraph.

In Data management and statistical analysis section in methods, "interquartile Range (IQR)" i in interquartile needs to be capitalized.

In socio-demographic characteristics of the participants in results, "majority (95.1%) of them were from Hindu religion" needs to be restructured to make it more coherent and fluent.

Behavioural and metabolic risk factors of CVDs in results: "Majority of the respondents were lifetime abstainer(79.7%). Current alcohol users 18.2%." bit confusing and need to be rephrased in a single sentence. In "Majority of the respondents 86.4%" write percent insides brackets. Need to correct spelling of "respiondents" in Metabolic risk factors.

Please mention the P-vlaues in AOR with 95% CI in "Determinant of metabolic risk factor". Mention statistically significant correlations at first and then non-significant ones.

In discussion: Instead of describing the prevalence of behavioural risk factors individually, it is better to write them together and correlate with their prevalence among cardiovascular disease patients, not with NCD risk factors survey findings as NCD is a umbrella term. Highlight more on the risk factors that are correlated significantly.

It is always good to point out some limitations that your study failed to address.

- **Correction done as per feedback.**

**Thank you**

**Suraksha Khatri**
